# DNA-dependent protein kinase catalytic subunit (DNA-PKcs)-SIN1 association mediates ultraviolet B (UVB)-induced Akt Ser-473 phosphorylation and skin cell survival

**DOI:** 10.1186/1476-4598-12-172

**Published:** 2013-12-24

**Authors:** Ying Tu, Chao Ji, Bo Yang, Zhi Yang, Hua Gu, Chun-Cheng Lu, Rong Wang, Zhong-Lan Su, Bin Chen, Wei-Ling Sun, Ji-Ping Xia, Zhi-Gang Bi, Li He

**Affiliations:** 1 Department of Dermatology, The First Affiliated Hospital of Kunming Medical University, Yunnan Provincial Institute of Dermatology, Kunming 650032, Yunnan, China; 2 Department of Dermatology, The First Affiliated Hospital of Nanjing Medical University, Nanjing 210024, Jiangsu, China; 3 Department of Dermatology, Longhua Hospital, Shanghai University of Traditional Chinese Medicine, 725 South Wanping Road, Shanghai 200032, China; 4 Key Laboratory of Reproductive Medicine, School of Public Health, Institute of Toxicology, Nanjing Medical University, Nanjing 210029, Jiangsu, China; 5 Laboratory of Reproductive Medicine, The Research Center for Bone and Stem Cells, Nanjing Medical University, Nanjing 210029, Jiangsu, China; 6 Department of Dermatology, BenQ Medical Center, Nanjing Medical University, Nanjing 210019, Jiangsu, China

**Keywords:** UV irradiation, Akt Ser-473 phosphorylation, DNA-PKcs, SIN1, Skin care

## Abstract

**Background:**

The exposure of skin keratinocytes to Ultraviolet (UV) irradiation leads to Akt phosphorylation at Ser-473, which is important for the carcinogenic effects of excessive sun exposure. The present study investigated the underlying mechanism of Akt Ser-473 phosphorylation by UVB radiation.

**Results:**

We found that DNA-dependent protein kinase catalytic subunit (DNA-PKcs) and mammalian target of rapamycin (mTOR) complex 2 (mTORC2) were both required for UVB-induced Akt Ser-473 phosphorylation in keratinocytes. Inhibition of DNA-PKcs activity via its inhibitor NU7026, a dominant-negative kinase-dead mutation, RNA interference (RNAi) or gene depletion led to the attenuation of UVB-induced Akt Ser-473 phosphorylation. Meanwhile, siRNA silencing or gene depletion of SIN1, a key component of mTORC2, abolished Akt Ser-473 phosphorylation by UVB. Significantly, we discovered that DNA-PKcs was associated with SIN1 in cytosol upon UVB radiation, and this complexation appeared required for Akt Ser-473 phosphorylation. Meanwhile, this DNA-PKcs-SIN1 complexation by UVB was dependent on epidermal growth factor receptor (EGFR) activation, and was disrupted by an EGFR inhibitor (AG1478) or by EGFR depletion. UVB-induced complexation between DNA-PKcs and mTORC2 components was also abolished by NU7026 and DNA-PKcs mutation. Finally, we found that both DNA-PKcs and SIN1 were associated with apoptosis resistance of UVB radiation, and inhibition of them by NU7026 or genetic depletion significantly enhanced UVB-induced cell death and apoptosis.

**Conclusion:**

Taken together, these results strongly suggest that DNA-PKcs-mTORC2 association is required for UVB-induced Akt Ser-473 phosphorylation and cell survival, and might be important for tumor cell transformation.

## Background

Skin cancers account for more than 30% of all newly diagnosed cancers around the world [1,2]. Solar Ultraviolet (UV) radiation, particularly its UVB component, is the major carcinogen for over 90% of all skin cancers [3]. UVB radiation causes cell DNA damage, along with activating of several signal transduction pathways, which regulate transcription of genes response for tumor initiation [4,5]. The majority of these initiated cells divide much faster than normal cells, and with colonel expansion and apoptosis evasion, these cells will transform into cancerous cells if not eliminated [1]. Among all signaling pathways activated by UVB irradiation, phosphoinositide 3-kinase (PI3K)/Akt/mammalian target of rapamycin (mTOR) pathway enhances the survival of mutated cells, thereby promoting skin cancer [6-8]. The survival-promoting function of PI3K/Akt after UVB irradiation results from the inhibition of caspases-3, -8, and −9 [6]. Thus, Akt signaling is a reasonable target for skin cancer prevention. As a matter of fact, our previous study has shown that perifosine, the Akt inhibitor, blocked UVB-induced Akt/mTOR activation, leading to a striking increase in skin cell apoptosis and a significantly reduced amount of DNA damages [9], and we suggested that perifosine might represent a novel agent for skin cancer prevention [9]. UVB-induced activation of Akt signaling has been shown to be dependent on epidermal growth factor receptor (EGFR) trans-activation [8]. However, how UVB activates Akt is still not fully understood.

DNA-dependent protein kinase (DNA-PK) is a nuclear serine/threonine protein kinase consisting of a 460-kDa catalytic subunit (DNA-PKcs) and the Ku heterodimer (Ku70 and Ku80) [10,11]. DNA-PKcs, belonging to PI3K-like protein kinase (PIKK), is one of the main kinases activated following UVB radiation [12,13]. It is critical for DNA double-strand break repair via the nonhomologous end joining (NHEJ) pathway [10,11]. It is known that UV radiation induces a rapid DNA-PKcs activation through phosphorylation [10,11]. Activation of DNA-PKcs by UV is dependent on ATR (Ataxia telangiectasia mutated and Rad3 related) kinase and is important for replication stress [12]. Interestingly, it is shown that DNA-PKcs is also important for Akt activation under certain stimuli [14,15]. The full activation of Akt requires phosphorylation on both Thr-308 and Ser-473 by 3-phosphoinositide-dependent kinase-1 (PDK1) and Ser-473 kinase, respectively. Although PDK1 has been well characterized, the signal mechanism that phosphorylates Akt at Ser-473 upon UVB radiation is still unknown. Recent studies have confirmed mTOR Complex 2 (mTORC2) [16] and DNA-PKcs [14,15] as potential Akt Ser-473 kinases. DNA-PKcs is found to directly associate and activate with Akt [14,15]. MTORC2, a complex consisting of mTOR, rapamycin-insensitive companion of mTOR (Rictor), mLST8, Protor, Deptor, and stress-activated protein kinase interacting protein 1 (SIN1) [17], is a major hydrophobic kinase that phosphorylates Akt on the Ser-473 (but not Thr-308) [18], which is required for Akt fully activation. Meanwhile, mTORC2 activation is also important for cell proliferation, survival, and nutrient uptake [19].

In light of these evidences, we hypothesized that DNA-PKcs could play a significant role in UVB-induced Akt activation. To test this hypothesis, we investigated whether UVB-activated DNA-PKcs could activate Akt and induce its phosphorylation, and the role mTORC2 in it. Our data showed that upon UVB irradiation, DNA-PKcs is activated and forms with a complex with mTORC2 component SIN1. This complexation appears required for Akt Ser-473 phosphorylation and cell survival. We indentified a link between DNA-PKcs and mTORC2 in Akt Ser-473 phosphorylation and cell survival upon UVB radiation.

## Results

### UVB activates Akt, mTORC1 and mTORC2 signalings in primary skin keratinocytes

**Figure 1:**
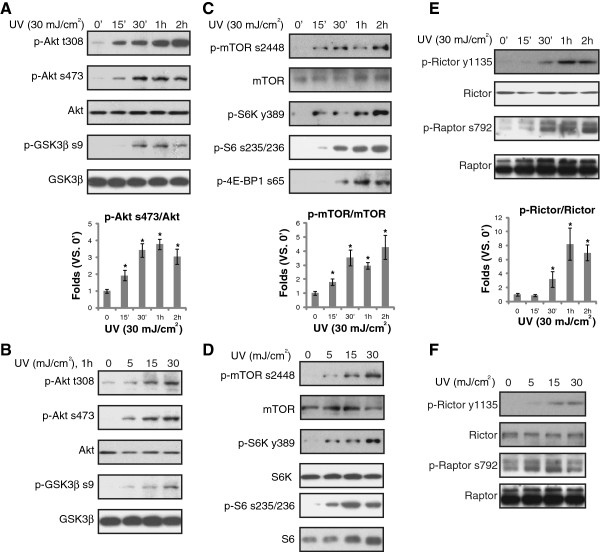
**UVB activates Akt, mTORC1 and mTORC2 signalings in primary skin keratinocytes.** Primary cultured human skin keratinocytes were exposed to UVB radiation (UV, 30 mJ/cm^2^) and cultured in DMEM for indicated time (0′, 15′, 30′, 1 hr and 2 hr) **(A, C and E)**, or irradiated with indicated concentration of UVB (0, 5, 15 and 30 mJ/cm^2^), and cultured for 1 hr **(B, D, F)**, activation of Akt, mTORC1 and mTORC2 in these cells was tested by immuno-blots using phosphorylation antibodies described. Non-phosphorylated kinases were also tested as loading controls. Phosphorylation (p) of Akt (Ser-473), mTOR (Ser 2448) and Rictor (Ser 792) was quantified. The values in the figures were expressed as the means ± standard deviation (SD) (Same for the following figures). All experiments were repeated three times and similar results were obtained (Same for the following figures). Statistical significance was analyzed by ANOVA (Same for the following figures).*p < 0.05 vs. 0′.

Activation of Akt/mTOR pathway is the major regulator of apoptosis resistance and cancer transformation in UVB irradiated skin cells [8,20-23]. It is known that mTOR kinase forms at least two distinct multi-protein complexes, namely mTOR complex 1 (mTORC1), or the rapamycin sensitive complex composed of mTOR, Raptor and PRAS40 [24], as well as mTOR mTORC2 [25-27]. mTORC1 phosphorylates S6K and 4E-BP1 [24]. MTORC2 promotes Akt Ser-473 phosphorylation, and increases its activity [19]. Thus, we tested Akt and mTOR activation in primary cultured skin keratinocytes. Akt activation was reflected by phospho(p)-Akt (Ser-473 and Thr-308) and its downstream p-GSK3β (Ser 9), mTORC1 activation was shown by p-S6K (Thr 389), p-S6 (Ser 235/236), p-4EBP1 (Ser 65) and p-Raptor (Ser 792). The activation of mTORC2 was confirmed by p-Akt (Ser-473) and p-Rictor (Tyr 1135). Immunoblotting results in Figure 1 showed that UVB radiation induced significant activation of Akt (Figure 1A and B), mTORC1 (Figure 1C-F) and mTORC2 (Figure 1A, B, E and F) in primary cultured skin keratinocytes. The effect of UVB was time- (Figure 1A, C and E) and dose-dependent (Figure 1B, D and E). Thus, our results confirmed that UVB activates Akt, mTORC1 and mTORC2 signalings in primary skin keratinocytes.

### DNA-PKcs activation is required for UVB-induced Akt Ser-473 phosphorylation

**Figure 2:**
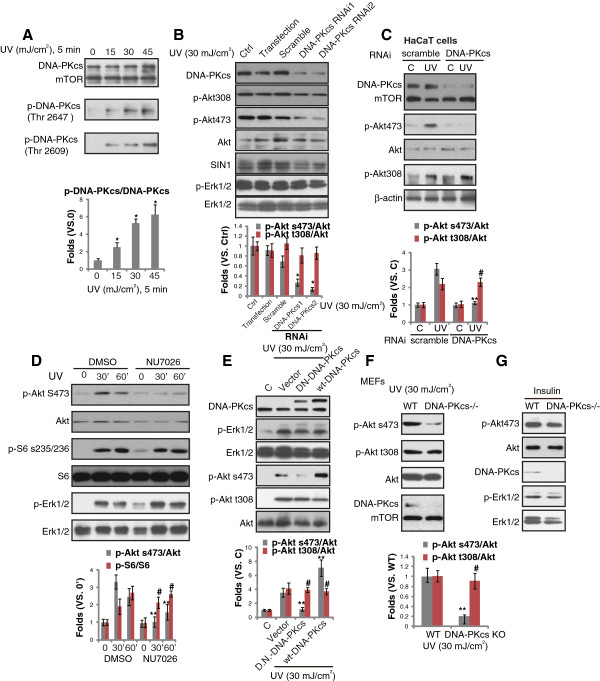
**DNA-PKcs activation is required for UVB-induced Akt Ser-473 phosphorylation.** UVB radiation induced DNA-PKcs phosphorylation in primary skin keratinocytes **(A)**, DNA-PKcs phosphorylation (Thr 2647) was quantified. UVB(UV, 30 mJ/cm^2^, 1 hr)-induced Akt Ser-473 and Thr-308 phosphorylation in primary skin keratinocytes transfected with scramble or two distinct DNA-PKcs siRNAs (“-1” was from Santa Cruz, “-2” was from Dharmacon) (100 nM each). Expressions of DNA-PKcs, SIN1 and p- and t- Akt/Erk were also tested **(B)**, Akt phosphorylation was quantified. HaCaT cells transfected with scramble or DNA-PKcs siRNA-2 (100 nM each) were left untreated or irradiated with UVB (UV, 30 mJ/cm^2^) for 1 hr, followed by immuno-blot detecting Akt (p- and regular), DNA-PKcs, mTOR and β-actin **(C)**, Akt phosphorylation was quantified. Effect of NU7026 (10 μM, 1 hr pretreatment) on UVB (UV, 30 mJ/cm^2^)-induced Akt, S6 and Erk1/2 phosphorylation was shown, regular Akt, Erk and S6 were also shown **(D)**, Akt and S6 phosphorylation was quantified. Primary keratinocytes, transfected with vector, dominant negative kinase dead DNA-PKcs (T2609A, DN) or WT-DNA-PKcs (1.0 μg/ml each), irradiated with UVB (UV, 30 mJ/cm^2^) for 1 hr, DNA-PKcs, Akt (p- and regular), Erk (p- and regular) were tested **(E)**, Akt phosphorylation was quantified. WT and DNA-PKcs KO MEFs were irradiated with UVB (UV, 30 mJ/cm^2^) for 1 hr, followed by immunoblotting detecting DNA-PKcs, Akt (p- and regular) and mTOR **(F)**, Akt phosphorylation was quantified. WT and DNA-PKcs KO MEFs were treated with insulin (200 nM) for 10 min, p- and t- Akt/Erk1/2 as well as DNA-PKcs expression were tested **(G)**. p < 0.05 vs. 0 or Ctrl (A and B). **p < 0.05 vs. control group **(C-F)**. ^**#**^p > 0.05 vs. control group **(C-F)**.

The key issue of this study is to understand the potential role of DNA-PKcs in UVB-induced Akt activation. As shown in Figure 2A, UVB induced DNA-PKcs activation in primary keratinocytes, which was demonstrated by its phosphorylation at Thr 2647 and Thr 2609 (Figure 2A). Importantly, DNA-PKcs siRNA knockdown significantly inhibited UVB-induced Akt Ser-473 phosphorylation, while leaving Akt Thr-308 phosphorylation unaffected (Figure 2B). We used two distinct, non-overlapping siRNAs against DNA-PKcs, and both siRNAs inhibited DNA-PKcs expression and Akt Ser-473 phosphorylation (Figure 2B). Similar results were also seen in transformed keratinocytes (HaCaT cells), as DNA-PKcs siRNA knockdown dramatically inhibited UVB-induced Akt Ser-473 (but not Thr-308) phosphorylation in HaCaT cells (Figure 2C). NU7026, the DNA-PKcs kinase inhibitor [28,29], almost abolished UVB-induced Akt Ser-473 phosphorylation (Figure 2D), indicating that DNA-PKcs kinase activity was required for Ser-473 phosphorylation by UVB. To further support this, we found that UVB-induced Ser-473 phosphorylation was suppressed by DNA-PKcs kinase-dead mutation (T2609A), while over-expression of WT-DNA-PKcs enhanced Ser-473 phosphorylation (Figure 2E). In both conditions, Akt Thr-308 phosphorylation was not affected (Figure 2E). Note that UVB-induced S6 phosphorylation, an indicator of mTORC1 activation, and Erk1/2 phosphorylation were not affected by NU7026 or DNA-PKcs mutation (Figure 2D and E). Thus, our results suggest that DNA-PKcs activation is required for UVB-induced Akt Ser-473 phosphorylation, which is further supported by the fact that DNA-PKcs knockout (KO) mouse embryonic fibroblasts (MEFs) showed a defective Akt Ser-473 phosphorylation after UVB radiation (Figure 2F). Note that Ku70 siRNA knockdown had almost no effect on UVB-induced Akt Ser-473 phosphorylation in cultured skin keratinocytes, suggesting that DNA-PKcs, but not Ku70 is required for UVB-induced Akt Ser 473 phosphorylation (Additional file 1: Figure S1). Further, insulin-induced Akt Ser-473 and Erk1/2 phosphorylation was not affected by DNA-PKcs deficiency in MEFs (Figure 2G).

### DNA-PKcs-SIN1 cytosol association mediates UVB-induced Akt Ser-473 phosphorylation

**Figure 3:**
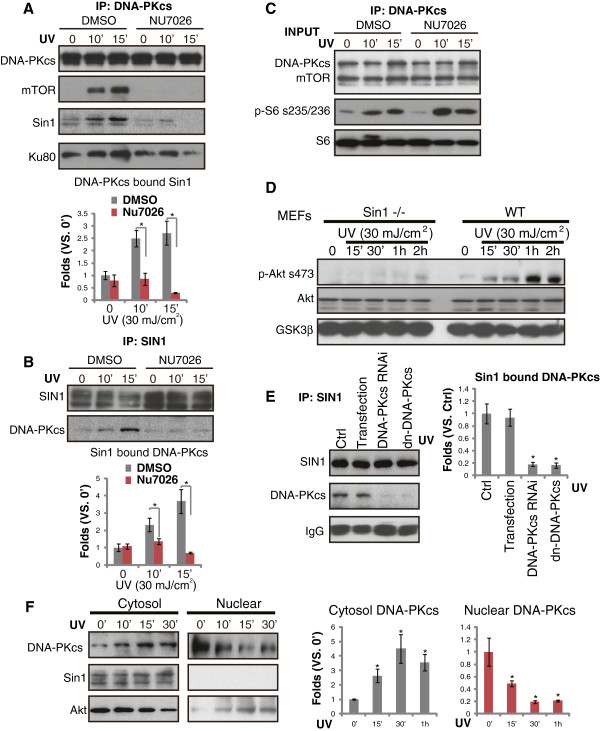
**DNA-PKcs-SIN1 cytosol association mediates UVB-induced Akt Ser-473 phosphorylation.** Effect of NU7026 (10 μM, 1 hr pretreatment) on UVB (UV, 30 mJ/cm^2^)-induced DNA-PKcs/SIN1/mTOR association was tested by Co-IP **(A and B)**, SIN1-DNA-PKcs association was quantified. The expression of DNA-PKcs/mTOR/SIN1 as well as S6 phosphorylation in whole cell lysates were also shown (**C**, input). WT and SIN1 KO (−/−) MEFs were irradiated with UVB (UV, 30 mJ/cm^2^) and cultured for 1 hr, followed by immunoblotting detecting p-Akt, regular Akt and GSK3β **(D)**. Primary skin keratinocytes, transfected with dominant negative (DN) DNA-PKcs, scramble siRNA or DNA-PKcs siRNA (“-2”), were irradiated with UVB (UV, 30 mJ/cm^2^) and cultured for 15 min, the association between SIN1/DNA-PKcs was tested by Co-IP **(E)**, SIN1 bound DNA-PKcs was quantified. Cytosol and nuclear DNA-PKcs, Akt and SIN1 in UVB (UV, 30 mJ/cm^2^) irradiated keratinocytes **(F)**, cytosol and nuclear DNA-PKcs was quantified. *p < 0.05 **(A)**, *p < 0.05 vs. 0′ or Ctrl **(E and F)**.

The above results suggested that DNA-PKcs is required for UVB-induced Akt Ser-473 phosphorylation. As discussed, mTORC2 is the known Ser-473 kinase. Thus, we tested whether a physical interaction between DNA-PKcs and mTORC2 components (i.e. SIN1 [30]) existed in UVB-treated cells. CoIP experiment results in Figure 3A and B confirmed DNA-PKcs-SIN1 association in UVB-irradiated keratinocytes, which was abolished by DNA-PKcs inhibitor NU7026 (Figure 3A and B). NU7026 had no affect on DNA-PKcs/mTOR expression or S6 phosphorylation (Figure 3C). Results from SIN1 KO MEFs confirmed that SIN1 was required for Akt Ser-473 phosphorylation by UV (Figure 3D). Notably, UVB-induced DNA-PKcs-SIN1 association was inhibited by DNA-PKcs mutation or silencing (Figure 3E), confirming the requirement of DNA-PKcs activity for the complexation. Importantly, DNA-PKcs was found to translocate from cell nuclei to cytosol after UVB radiation, while SIN1 stayed in the cytosol (Figure 3F). Thus, the binding between DNA-PKcs and SIN1, and the following Akt phosphorylation (Ser-473) were likely happening in the cytosol, although a small fraction of Akt was also found to shuttle into nuclear after UVB radiation (Figure 3F).

### EGFR activation is required for UVB-induced DNA-PKcs/SIN1 association and Akt phosphorylation (Ser-473)

**Figure 4:**
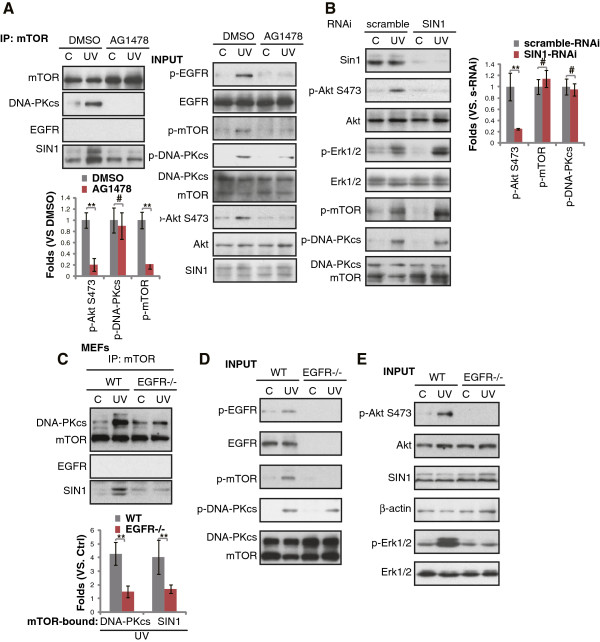
**EGFR activation is required for UVB-induced DNA-PKcs/SIN1 association and Akt phosphorylation (Ser-473).** Effect of NU7026 (10 μM, 1 hr pretreatment) on UVB (UV, 30 mJ/cm^2^, 15 min)-induced DNA-PKcs/SIN1/mTOR association was tested by Co-IP **(A)**, EGFR (p- and regular), mTOR (p- and regular), DNA-PKcs (p-Thr 2609, and regular), Akt (p- and regular) and Erk (p- and regular) in whole cell lysates were also tested (**A**, input). SIN1 knockdown in keratinocytes had no effect on UVB (UV, 30 mJ/cm^2^, 15 min)-induced DNA-PKcs/Erk/mTOR phosphorylation, but blocked Akt Ser 473 phosphorylation **(B)**, Erk, mTOR and DNA-PKcs phosphorylation was quantified. WT and EGFR KO MEFs were irradiated with UVB (UV, 30 mJ/cm^2^) for 15 min, followed by CoIP detecting of DNA-PKcs/SIN1 association, mTOR-bound DNA-PKcs and SIN1 was quantified; EGFR (p- and regular), mTOR (p- and regular) and expression of DNA-PKcs (p-Thr 2609, and regular) **(D)** as well as Erk (p- and regular), Akt (p- and regular), SIN1 and β-actin in whole cell lysates were tested **(E)**, **p < 0.05. **(B and C)**. ^**#**^p > 0.05 **(B)**.

Trans-activation of EGFR by UVB mediates activation of Akt and other signaling pathways [7,8]. We then wanted to know if EGFR was also important for UVB-induced DNA-PKcs/SIN1 association. As expected, AG1478, the EGFR inhibitor blocked UVB-induced mTOR activation (Ser 2448 phosphorylation) and Akt phosphorylation (Ser-473) (Figure 4A input). Importantly, UVB-induced DNA-PKcs/SIN1/mTOR association was also blocked (Figure 4A). Intriguingly, EGFR was not in the complex of DNA-PKcs/SIN1/mTOR (Figure 4A), suggesting that EGFR mediated DNA-PKcs/SIN1 complexation was an indirect effect. Further, EGFR depletion abolished UVB-induced DNA-PKcs/SIN1/mTOR association (Figure 4C), mTOR phosphorylation (Figure 4B) and Akt/Erk activation in MEFs (Figure 4E). However, UVB-induced DNA-PKcs activation (phosphorylation) was not affected by either AG1478 or EGFR depletion (Figure 4A and D), thus UVB-induced DNA-PKcs activation was not dependent on EGFR. SIN1 siRNA knockdown had no effect on UVB-induced DNA-PKcs and mTOR phosphorylation, suggesting that SIN1 likely lies downstream of DNA-PKcs (Figure 4B).

### DNA-PKcs/SIN1 inhibition exacerbates UVB-induced apoptosis

**Figure 5:**
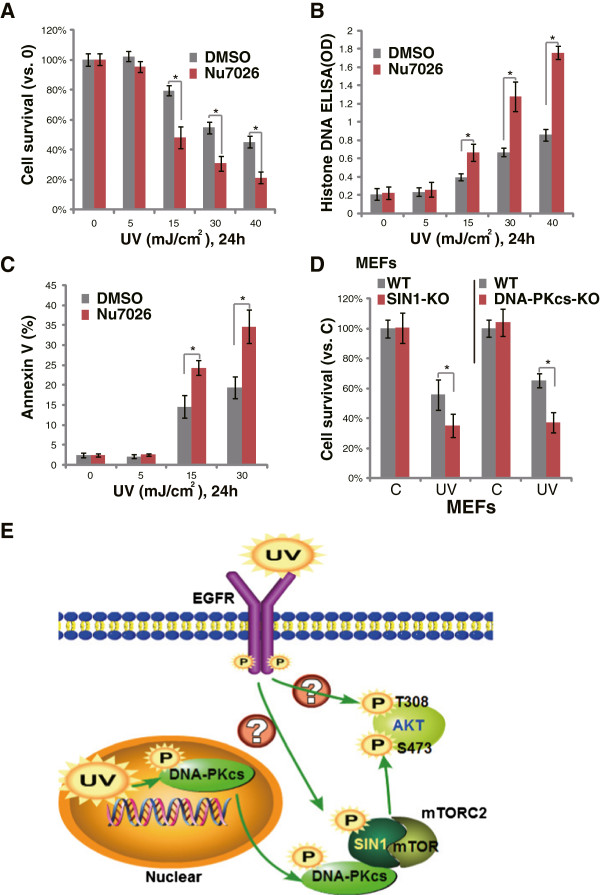
**DNA-PKcs inhibition enhanced UVB-induced apoptosis of keratinocytes.** Effect of NU7026 (10 μM, 1 hr pretreatment) on UVB (UV, 30 mJ/cm^2^)-induced cell viability loss and apoptosis were tested by MTT assay **(A)** and Histone-DNA ELISA **(B)**/Annexin V FACS assay **(C)** respectively. WT, DNA-PKcs KO or SIN1 KO MEFs were irradiated with UVB (UV, 30 mJ/cm^2^), cells were further cultured for 24 hrs, before cell viability was tested **(D)**. **(E)** The proposed signaling mechanism of the current study (see above discussion). *p < 0.05.

As shown in our previous study, activation of Akt by UVB radiation contributes to apoptosis-resistance and promotes survival of damaged cells [9]. The Akt inhibitor perifosine significantly enhances UVB-induced skin cell apoptosis and reduces DNA damages through Akt in-activation along with other actions [9]. Since DNA-PKcs mediates UVB-induced Akt Ser-473 phosphorylation, we then tested whether DNA-PKcs activation was also important for UVB-induced anti-apoptosis effect. As shown in Figure 5A, the DNA-PKcs kinase inhibitor NU7026 enhanced UVB-induced death of keratinocytes, which was shown by the decreased cell viability. Meanwhile, UVB-induced apoptosis was significantly enhanced by NU7026 (Figure 5B and C). These results suggested that DNA-PKcs activation is important for apoptosis resistance against UV. The fact that DNA-PKcs depletion also enhanced UVB-induced MEFs death (Figure 5D) further supported our proposal. Notably, SIN1 KO cells showed similar phenotype as DNA-PKcs KO cells, and were more sensitive to UVB-induced death (Figure 5D). Thus, DNA-PKcs/SIN1 inhibition exacerbates UVB-induced cell death and apoptosis.

## Discussion

The PI3K-like protein kinases DNA-PKcs along with ATM (Ataxia telangiectasia mutated) and ATR are the main kinases activated by various DNA assaults [11]. Although ATM and DNA-PKcs kinases are activated mainly upon DNA double-strand breaks, i.e. ionizing radiation (IR) [11], these kinases could also be rapidly phosphorylated and activated by ATR kinase upon UV irradiation [12], which mainly generates single strand breaks. DNA-PKcs is phosphorylated by UV irradiation in a pattern distinct from that induced by IR [12]. DNA-PKcs is rapidly phosphorylated at Thr 2609 and Thr 2647, but not at Ser 2056 (the IR site) after UV irradiation [12]. In the current study, we also observed DNA-PKcs phosphorylation at Thr 2609 and Thr 2647 after UVB radiation. More importantly, we found that DNA-PKcs mediates UVB-induced Akt Ser 474 phosphorylation and apoptosis resistance through complexation with the mTORC2 component SIN1 (see Figure 5E).

Identification of the kinases responsible for phosphorylating Akt at Ser-473 has been a major challenge for a number of years. It is known that Akt Ser-473 could be phosphorylated by both mTORC2 [16,27] and DNA-PKcs [14,15] depending on type of stimuli. Our previous studies have shown that siRNA knocking-down of mTOR or Rictor, two other key components of mTORC2 [16], blocked UVB-induced Akt Ser-473 phosphorylation [9]. In consist with these findings, we here found that UVB-induced Akt Ser-473 phosphorylation was abolished by SIN1 deficiency, thus mTORC2 is the Akt Ser 473 kinase for UVB. Importantly, DNA-PKcs was found to form a complex with SIN1 at the cytosol after UVB radiation, and our evidence suggested that this complexation might be critical to phosphorylate Akt at Ser-473. Thus, mTORC2 and DNA-PKcs were both required for Akt Ser-473 phosphorylation upon UVB radiation (see Figure 5E).

DNA-PKcs is a member of the PI3K-like kinase family. Available evidence indicates that DNA-PKcs has protein kinase activity, and can target downstream events of the PI3K pathway [14,31]. Although DNA-PKcs has been long proposed as a Ser-473 kinase [14,31], how DNA-PKcs phosphorylates Akt is still controversial. The physiological role of DNA-PKcs in the regulation of Akt phosphorylation remains to be established. Feng et al., showed that DNA-PKcs co-localizes and associates with Akt (PKB) at the plasma membrane, where DNA-PKcs directly phosphorylates Akt on Ser-473, resulting in a 10-fold enhancement of Akt activity [14]. While Chu et al., showed that DNA-PKcs associates with Akt upon CpG-DNA stimulation mainly in the cytosol, where DNA-PKcs phosphorylates Akt at both Ser-473 and Thr-308, and triggers Akt nuclear translocation [15]. DNA-PKcs is present mainly in the nucleus, but with a substantial amount also in the cytosol [14,32,33], whereas Akt mainly locates in cytosol. We found that many DNA-PKcs was also present in the cytosol, consistent with a previous observation that DNA-PKcs localization in lipid rafts [34]. Interestingly, we found that UVB radiation promoted nuclear DNA-PKcs translocation to cytosol. Since SIN1 was only present in the cytosol, we propose that upon UVB radiation, activated DNA-PKcs will translocate to cytosol, where it forms a complex with SIN1, which phosphorylates Akt at Ser-473 (see Figure 5E).

In the current study, we found that DNA-PKcs and mTORC2 work together to phosphorylate Akt at Ser-473 under UVB radiation. While UVB-induced Akt Thr-308 phosphorylation was not affected by DNA-PKcs or mTOR deficiency, suggesting that DNA-PKcs-mTORC2 is not required for Akt Thr 308 phosphorylation. Thus, the potential upstream kinase for Akt Thr 308 phosphorylation by UVB still needs to be identified. We found that UVB-induced Akt 308 phosphorylation was largely inhibited by EGFR inhibitor AG1478 (data not shown, also seen [8]), suggesting that 308 kinase is likely lying downstream of EGFR to phosphorylate Akt (Thr 308) upon UVB radiation. One possible kinase is PDK1 [35]. PDK1 is thought to play a central role in activating Akt and other AGC protein kinases [35], it works as the Thr 308 kinase and phosphorylates Akt at Thr 308 under growth factor or other stimulations [36,37]. Meanwhile, PDK1 can be activated by UVB [36]. However, how UVB-activated EGFR possibly activate PDK1, and how PDK1 phosphorylates Thr 308 under UVB radiation remain largely obscure (Figure 5E).

UVB radiation causes keratinocytes damage, and these cells respond to this damage in three ways: either by tolerating the damage, by repairing the damage through cell cycle suspension, or by undergoing apoptosis [38,39]. Apoptosis represents the defense mechanism to eliminate the defective or damaged cells [38,39]. However, those unrepaired cells with genetic abnormalities may undergo a colonial expansion to acquire a hyper-proliferative phenotype causing neoplastic transformation [40]. Although UVB radiation mainly activates pro-apoptotic signalings, it simultaneously activates anti-apoptotic kinases (i.e. Akt and NFκB) to promote survival of transformed (pre-cancer) cells [38,39]. In light of this, we found that DNA-PKcs and SIN1 were both important for keratinocytes survival after UVB radiation, and DNA-PKcs inhibition or SIN1 depletion enhanced UVB-induced cell death and apoptosis.

## Conclusion

In conclusion, our data suggested that UVB-activated DNA-PKcs forms a complex with mTORC2 component SIN1, which serves as Akt Ser 473 kinase and promotes cell survival.

## Methods

### Chemicals and reagents

NU7026 and AG1478 were purchased from Calbiochem (Shanghai, China). Annexin V apoptosis kit was obtained from Promega (Shanghai, China).Insulin, 3-(4,5-dimethyl-thiazol-2-yl)2,5-diphenyl tetrazolium bromide (MTT) dye was purchased from Sigma (Shanghai, China).

### Antibodies

Antibodies again Erk1/2, S6, Ku70, p-DNA-PKcs (Thr 2609) and Akt (1/2) were purchased from Santa Cruz Biotechnology (Santa Cruz, CA). Mouse anti-β-actin antibody was obtained from Sigma (Shanghai, China). Antibodies against phospho(p)-Akt (Ser 473), p-Akt (Thr 308), p-mTOR (Ser 2448), mTOR, p-S6K (ribosomal p70 S6 kinase 1,Thr 389), S6K, p-S6 (Ser 235/236), p-GSK3β (Ser 9), GSK3β, p-4E-BP1 (Ser 65), DNA-PKcs, EGFR, p-EGFR (Tyr 1068), Rictor, p-Rictor (Tyr 1135), Raptor, p-Raptor (Ser 792) were purchased from Cell Signaling Technology (Denver, MA). Anti-p-DNA-PKcs (Thr 2647) antibody was purchased from Abcam (Shanghai, China).

### Cell culture and UVB radiation

Primary human epidermal keratinocytes, HaCaT cells as well as various mouse embryonic fibroblasts (MEFs) were maintained in DMEM medium supplemented with a 10% FBS, with anti-biotic in a CO_2_ incubator at 37°C. UVB radiation equipments and procedure were described in [41,42]. EGFR wild-type (WT) and knockout (KO) MEFs were reported [7]. DNA-PKcs WT and DNA-PKcs MEFs were from Dr. Yang’s Lab [43]. SIN1 WT and SIN1 KO MEFs were gifts from Dr. Li at Nanjing Medical University.

***Western blot and cell viability (MTT) assay*** were described in our previous studies [44,45]. Nuclear and cytosol fraction of cells were isolated through the nuclear and cytosol separation kit purchased from Nanjing Kai-ji Biotech (Nanjing, China) according to the protocols. Band intensity was quantified with ImageQuant V5.1 software, and was normalized to control group (Molecular Dynamics, Piscataway, NJ).

### Quantification of apoptosis by ELISA

The Cell Apoptosis Histone-DNA ELISA PLUS Kit (Roche, Palo Alto, CA) was used to detect apoptosis in skin cells after different treatments as previously described [44,45].

### Apoptosis assay

After indicated treatment/s, keratinocytes were washed with cold PBS and incubated with the binding buffer attached, containing 2 μg/ml of annexin V-FITC for 10 min. Cells were then washed with PBS and resuspended. A total of 15,000 cells of each event were analyzed by flow cytometry (Beckton Dickinson FACScan). Annexin V (apoptotic) percentage was recorded.

### SiRNA

DNA-PKcs-siRNA-1 was purchased from Santa Cruz (sc-35200 h). DNA-PKcs-siRNA-2 (leading strand: GAUCGCACCUUACUCUGUUdTdT), targeting 352 bases downstream from the start codon, was purchased from Dharmacon Research Inc [46]. Scramble siRNA, Ku 70 siRNA (sc-29383) and SIN1 (MAPKAP1) siRNA (sc-60984) were also purchased from Santa Cruz. SiRNA transfection was performed through Lipofectamine 2000 (Invitrogen, Carlsbad, CA) according to recommendation procedure, see [44].

### Plasmids and transfection

A 3-kb *Hind*III fragment of DNA-PKcs cDNA covering Thr 2609 was used as the template for generating the T2609A mutation of DNA-PKcs cDNA. Site-directed mutagenesis was performed using the Quik Change site-directed mutagenesis kit (Stratagene) and the forward (TCCGATTTTGTGGAGsGACCAGGCCTCCCAGGGC) and reverse (GCCCTG GGAGGCCTGGTCCTCCACAAACATCGGA) primers [47]. The mutated DNA-PKcs cDNA fragment was assembled back into the full-length DNA-PKcs cDNA as described previously [47]. T2609A DNA-PKcs or the WT-DNA-PKcs cDNA were introduced into pSV2 neo plasmid, which was transfected into the keratinocytes through Lipofectamine 2000 protocol [44]. Forty-eight hours after transfection, cells were re-plated on selection medium containing 500 μg/mL of G418 for 8–10 days. Individual colonies were isolated and further characterized for expression.

### Co-immunoprecipitation (CoIP)

As previously reported [45], keratinocytes or MEFs with indicated treatments were lysed with lysis buffer containg 0.3% CHAPS and protease inhibitor cocktails (Roche Diagnostics, Indianapolis, IN). Aliquots of 1000 μg of proteins from each sample were pre-cleared by incubation with 15 μl of protein A/G Sepharose (beads) (Amersham, IL) for 1 hour at 4°C. Precleared samples were incubated with specific antibody (1–2 μg/sample) in lysis buffer (1000 μl) overnight at 4°C. To this was added 30 μl of protein A/G beads and the samples were incubated for 2 hours at 4°C. The beads were washed five times with phosphate-buffered saline (PBS) and once with lysis buffer, boiled, separated by 10% SDS-PAGE, and transferred onto a PVDF membrane followed by immunoblotting analysis.

### Statistical calculation

Each experiment was repeated a minimum of three times. In each experiment, the mean value of the repetitions was calculated and this value was used in the statistical analysis. All data were normalized to control values of each assay and were presented as mean ± SD. Data were analyzed by one-way ANOVA followed by a Scheffe’s f-test by using SPSS 15.0 software (SPSS Inc., Chicago, IL, USA). Significance was chosen as *p* < 0.05.

## Abbreviations

UVB: Ultraviolet B; mTOR: Mammalian target of rapamycin; mTORC1: mTOR Complex 1; mTORC2: mTOR Complex 2; DNA-PKcs: DNA-dependent protein kinase catalytic subunit; RNAi: RNA interference; EGFR: Epidermal growth factor receptor; S6K: Ribosomal p70 S6 kinase 1; eIF4E: Eukaryotic initiation factor 4E; 4E-BP1: Binding protein 1; siRNA: Small interfering RNA; MEFs: Mouse embryonic fibroblasts; KO: Knockout; MTT: 3-(4,5-Dimethylthiazol-2-yl)-2,5-diphenyltetrazolium bromide.

## Competing interests

The authors declare that they have no competing interests.

## Authors’ contributions

YT, BY, ZY, HG, CL, RW, ZS, BC, WS and JX carried out the experiments. YT, BY, ZY, CJ, ZB and LH participated in the design of the study and performed the statistical analysis. YT, BY, ZY, CJ, ZB and LH conceived of the study, and participated in its design and coordination and helped to draft the manuscript. All authors read and approved the final manuscript.

## Supplementary Material

Additional file 1: Figure S1 UVB-induced Akt S473 phosphorylation is not affected by Ku 70 siRNA knockdown. Primary skin keratinocytes, transfected with scramble siRNA or Ku 70 siRNA, were either left untreated (“C”) irradiated with UVB (UV, 30 mJ/cm^2^) and cultured for 15 min, p-/t- Akt and Erk1/2 as well as Ku 70 expression were tested by western blots. Click here for file
