# Supplementary figures and images for: DNA-dependent protein kinase catalytic subunit (DNA-PKcs)-SIN1 association mediates ultraviolet B (UVB)-induced Akt Ser-473 phosphorylation and skin cell survival
Source: Mol Cancer. 2013 Dec 24;12:172. doi: 10.1186/1476-4598-12-172 (PMC3922905; doi:10.1186/1476-4598-12-172)

# Figure S1

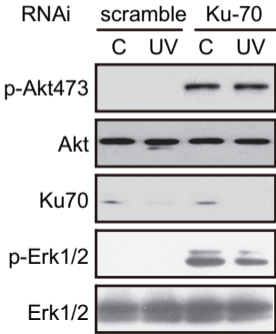

Supplement: Additional file 1: Figure S1 — UVB-induced Akt S473 phosphorylation is not affected by Ku 70 siRNA knockdown. Primary skin keratinocytes, transfected with scramble siRNA or Ku 70 siRNA, were either left untreated (“C”) irradiated with UVB (UV, 30 mJ/cm2) and cultured for 15 min, p-/t- Akt and Erk1/2 as well as Ku 70 expression were tested by western blots. [file 1476-4598-12-172-S1.pdf]
